# Impact of scanning parameters and breathing patterns on image quality and accuracy of tumor motion reconstruction in 4D CBCT: a phantom study

**DOI:** 10.1120/jacmp.v16i6.5620

**Published:** 2015-11-08

**Authors:** Soyoung Lee, Guanghua Yan, Bo Lu, Darren Kahler, Jonathan G. Li, Samant S. Sanjiv

**Affiliations:** ^1^ J. Crayton Pruitt Family Department of Biomedical Engineering University of Florida Gainesville FL USA; ^2^ Department of Radiation Oncology College of Medicine, University of Florida Gainesville FL USA

**Keywords:** image quality, four‐dimensional cone‐beam CT (4D CBCT), registration, motion trajectory, image‐guided radiation therapy (IGRT)

## Abstract

Four‐dimensional, cone‐beam CT (4D CBCT) substantially reduces respiration‐induced motion blurring artifacts in three‐dimension (3D) CBCT. However, the image quality of 4D CBCT is significantly degraded which may affect its accuracy in localizing a mobile tumor for high‐precision, image‐guided radiation therapy (IGRT). The purpose of this study was to investigate the impact of scanning parameters (hereinafter collectively referred to as scanning sequence) and breathing patterns on the image quality and the accuracy of computed tumor trajectory for a commercial 4D CBCT system, in preparation for its clinical implementation. We simulated a series of periodic and aperiodic sinusoidal breathing patterns with a respiratory motion phantom. The aperiodic pattern was created by varying the period or amplitude of individual sinusoidal breathing cycles. 4D CBCT scans of the phantom were acquired with a manufacturer‐supplied scanning sequence (4D‐S‐slow) and two in‐house modified scanning sequences (4D‐M‐slow and 4D‐M‐fast). While 4D‐S‐slow used small field of view (FOV), partial rotation (200°), and no imaging filter, 4D‐M‐slow and 4D‐M‐fast used medium FOV, full rotation, and the F1 filter. The scanning speed was doubled in 4D‐M‐fast (100°/min gantry rotation). The image quality of the 4D CBCT scans was evaluated using contrast‐to‐noise ratio (CNR), signal‐to‐noise ratio (SNR), and motion blurring ratio (MBR). The trajectory of the moving target was reconstructed by registering each phase of the 4D CBCT with a reference CT. The root‐mean‐squared‐error (RMSE) analysis was used to quantify its accuracy. Significant decrease in CNR and SNR from 3D CBCT to 4D CBCT was observed. The 4D‐S‐slow and 4D‐M‐fast scans had comparable image quality, while the 4D‐M‐slow scans had better performance due to doubled projections. Both CNR and SNR decreased slightly as the breathing period increased, while no dependence on the amplitude was observed. The difference of both CNR and SNR between periodic and aperiodic breathing patterns was insignificant (p>0.48). At end‐exhale phases, the motion blurring was negligible for both periodic and aperiodic breathing patterns; at mid‐inhale phase, the motion blurring increased as the period, the amplitude or the amount of cycle‐to‐cycle variation on amplitude increased. Overall, the accuracy of localizing the moving target in 4D CBCT was within 2 mm under all studied cases. No difference in the RMSEs was noticed among the three scanning sequences. The 4D‐M‐fast scans, free of volume truncation artifacts, exhibited comparable image quality and accuracy in tumor motion reconstruction as the 4D‐S‐slow scans with reduced imaging dose (0.60 cGy vs. 0.99 cGy) due to the use of faster gantry rotation and the F1 filter, suggesting its suitability for clinical use.

PACS number: 87.55.Qr

## I. INTRODUCTION

Three‐dimensional (3D) cone‐beam computed tomography (CBCT) has become the primary image guidance technique in stereotactic body radiation therapy (SBRT) for lung cancer.^1^, ^2^ Volumetric imaging acquired using CBCT provides the location and orientation of patient anatomy in the treatment coordinate system.^(3)^ The ability to distinguish lung, soft tissue, and bony anatomies in 3D makes CBCT technique suitable for intensity‐based registration with six degrees of freedom, providing target registration accuracy superior to that of two‐dimensional (2D) or megavoltage imaging.^4^, ^5^, ^6^, ^7^, ^8^ Daily online correction with CBCT prior to treatment can consequently improve patient positioning accuracy.^6^, ^9^ However, due to the limited gantry rotation speed ($∼6^{\circ }/s$), CBCT images inevitably suffer from respiration‐induced motion artifacts, particularly in the thoracic and upper‐abdominal region of the patient. These artifacts can introduce significant uncertainties in high‐precision, image‐guided radiation therapy (IGRT).^(10)^

Respiration‐correlated or four‐dimensional CBCT (4D CBCT) imaging technique images organ motions corresponding to individual breathing phases,^11^, ^12^ potentially overcoming the aforementioned limitations of 3D CBCT. Four‐dimensional CBCT has been used to determine time‐averaged tumor position and amplitude change of tumor motion and to validate treatment margins by accounting for inter‐ and intrafractional variability from a full set of respiratory phases.^12^, ^13^, ^14^ This technology can provide benefits to lung cancer patients by allowing for accurate tumor localization and reliable treatment delivery with adequate tumor coverage.^10^, ^11^, ^12^, ^13^, ^14^, ^15^

Although promising, 4D CBCT suffers from longer image acquisition time and poorer image quality than 3D CBCT, which hinders its widespread use in daily clinical practice.^16^, ^17^ In 4D CBCT, projections are acquired over multiple respiratory cycles with slow gantry rotation. These projections are then retrospectively sorted into multiple respiratory bins according to different motion phases estimated from a respiratory signal. The respiratory binning process can result in large angular spacing between consecutive projections of the same respiratory phase and projection clustering within the same phase, leading to severe view‐aliasing artifacts such as strong streaks in the phased 4D CBCT images. To alleviate the problem, 4D CBCT utilizes longer scan times to increase the number of projections. Consequently, to maintain a similar radiation dose level to the patient as conventional CBCT, reduced exposure (mAs/projection) is typically used in 4D CBCT. However, reducing exposure decreases signal‐to‐noise ratio in the projections, thereby further degrading the image quality. Thus, there is a tradeoff between image quality, image dose, and image acquisition time in 4D CBCT. A balance needs to be reached by optimizing scanning parameters (such as gantry rotation speed and range, mAs/projection) to make 4D CBCT a viable tool for image‐guided SBRT.

Besides the scanning parameters, patient breathing pattern could significantly affect the image quality in 4D CBCT. The respiratory binning process relies on detecting the position of a distinct moving object (e.g., the diaphragm or tumor itself) in the projection view to decide when a projection is acquired within the patient's breathing cycle. The current 4D CBCT imaging technique employs a constant frame rate (frames per second) and a constant gantry rotation speed.^(18)^ Thus, a regular breathing pattern will lead to relatively evenly spaced projections, while an irregular or erratic breathing pattern could significantly worsen the evenness of projection distribution among individual phases and projection clustering within phases, which in turn degrades image quality.^19^, ^20^ The degradation in image quality could reduce image registration accuracy and potentially lead to additional uncertainty in patient setup, hindering the clinical acceptance of 4D CBCT as an effective image‐guidance tool.

Since Sonke et al.^(12)^ reported the method to generate respiratory‐corrected CBCT, a great deal of research effort has been devoted to developing new strategies for 4D CBCT, with the goal of reducing scanning time and imaging dose while improving image quality.^15^, ^19^ Shieh et al.^(18)^ studied the impact of controllable factors, including the source of respiratory signal, binning methods, and reconstruction algorithms, on the image quality of 4D CBCT and concluded that, with current technique limits, the most effective strategy for improving image quality was to develop a better reconstruction algorithm. To reduce projection clustering and achieve more uniform projection distribution, Cooper et al.^(19)^ proposed respiratory‐triggered 4D CBCT which utilized respiratory signal to trigger the projection acquisitions. Their simulation suggested a 47% reduction in imaging dose with negligible degradation in image quality. In the approach of Li and Xing,^(15)^ gantry rotation speed was customized in accordance with patient's breathing pattern for better image quality. Methods to correct for respiratory‐induced motion artifacts using *a priori* motion models and patient‐specific motion models have been reported by Rit et al.^(21)^ and Zhang et al,^(22)^ respectively. In the development of compressed sensing‐based iterative reconstruction algorithms for 4D CBCT, several groups reported superior image quality with significantly reduced number of projections (thus imaging dose).^23^, ^24^, ^25^ It is worth noting that these studies are either retrospective or implemented within research settings. On commercially available 4D CBCT systems, the clinic impact of these new strategies remains to be seen.

A commercial 4D CBCT system, Elekta Symmetry (XVI R4.5; Elekta Oncology System Ltd., Crawley, UK), recently became available in our institution. In preparation for its clinical use, a thorough understanding of its performance (image quality and accuracy of tumor motion trajectory reconstruction) was needed. Sweeney et al.^(17)^ reported a comparison study on this system using 3D and 4D CBCT for lung SBRT, with encouraging results for 4D CBCT in more precise target localization. However, a comprehensive study on the impact of scanning parameters and irregular breathing patterns on the performance of the system does not exist yet. A detailed analysis of various imaging sequences will enable us to make adjustments based on assessments, thereby aiding the utilization of optimal image acquisition parameters.

This work seeks to investigate the effects of the scanning parameters and respiratory motion characteristics on 4D CBCT image quality and accuracy of computed tumor trajectory in lung SBRT. We simulated various respirations (periodic and aperiodic sinusoidal patterns with varying periods and amplitudes) with a commercial respiratory motion phantom (Quasar, Modus Medical Device, Inc., London, Canada). Four‐dimensional CBCT scans of the Quasar phantom were acquired with three scanning sequences (one vendor‐recommended and two in‐house‐modified) by varying scanning parameters such as field of view (FOV) and gantry rotation speed. Image quality of the 4D CBCT was assessed for each respiratory phase with the lung tumor target in the Quasar phantom. Using an automatic image registration method, we reconstructed tumor motion trajectories from the 4D CBCT images of the lung tumor target. The accuracy of the extracted target motion trajectories was evaluated with respect to the reference respiratory motion programmed on the phantom. The results of this study can help us understand the relationship between varying breathing patterns and the image quality of a commercial 4D CBCT system, and optimize the scanning sequence to achieve clinically acceptable image quality for patient localization while minimizing imaging dose and the scanning time.

## II. MATERIALS AND METHODS

### A. Tumor motion design with a respiratory phantom

**Figure 1:**
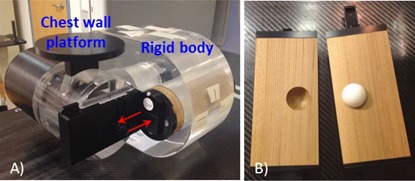
The Quasar respiratory motion phantom (a) and the moving rod with a target ball (b). The rod moves along the superior–inferior (SI) direction (indicated by the arrows).

The Quasar phantom was used to simulate patient breathing pattern. Various respiration patterns were designed and applied to the moving insert in the phantom. A spherical target ball ($1.05\;g/{cm}^{3}$, 30 mm in diameter) embedded in a rod with density equivalent to that of lung ($0.04\;g/{cm}^{3}$) mimicked a moving lung tumor. The rod moved along the superior–inferior (SI) direction (indicated by the arrows in Fig. 1) within the body phantom according to the respiration pattern programmed in the controller.

To investigate the influence of breathing irregularity on image quality and accuracy of motion reconstruction in 4D CBCT, a series of periodic and aperiodic respiratory motions were simulated and applied on the Quasar phantom. For simplicity, a periodic sinusoidal waveform was assumed as a representative respiratory motion pattern, which is described as:

(1) x(iΔt)=a sin(2πiΔt/T)

here i=1,2,3,…,x(iΔt) is the target position at time iΔt with the sampling interval Δt set at 0.01 s, and α and *T* are the amplitude and period of the motion, respectively. As several studies have shown,^13^, ^26^, ^27^ the peak‐to‐peak amplitude of patient tumor motion can be as large as 30 mm, and the breathing period typically varies from 3 to 6 s. Thus, we simulated respiratory motion with peak‐to‐peak amplitude of 10, 20, and 30 mm, and period of 3, 4, 5, and 6 s.

In reality, a patient's respiratory motion can exhibit variations in amplitude and period during 4D CBCT acquisition. To avoid confusion in this article, sinusoidal motion patterns with cycle‐to‐cycle variations in either amplitude or period are referred to as aperiodic sinusoidal motion patterns, which could be generated by varying the amplitude or period of individual breathing cycles of a periodic sinusoidal pattern, as described in Ruan et al.^(28)^ To separately evaluate the effect of cycle‐to‐cycle variability of period and amplitude on image quality, two different forms of aperiodic sinusoidal respirations were generated using the basic form of the sine wave function:

(2) $$x(i\Delta t)=a\text{sin}(2\pi i\Delta t/T_{n}),\;f\;or\;\sum_{j=1}^{n-1}T_{j}/\Delta t<i\leq \sum_{j=1}^{n}T_{j}/\Delta t;$$

(3) $$x(i\Delta t)=a_{n}\;\text{sin}(2\pi i\Delta t/T),\;f\;or\;\sum_{j=1}^{n-1}T_{j}/\Delta t<i\leq \sum_{j=1}^{n}T_{j}/\Delta t.$$

where $T_{n}$ and $a_{n}$ are the period and amplitude of the nth respiratory cycle, sampled from normal distribution, $Ν(T,\sigma_{T}^{2})$ and $Ν(\alpha ,\sigma_{a}^{2})$, respectively. Four mean period values (T=3,4,5 and 6 s) with two relative standard deviations ($\left(\sigma_{T}=0.3\;\text{and}\;0.7\right)$) and three mean amplitude values (α=10,20, and 30 mm) with two relative standard deviations (SDs) ($\sigma_{a}=0.06\;\text{and}\;0.16$) were used in this study. These values were chosen based on a survey of published observations on lung motion.^26^, ^29^, ^30^

Additionally, the breathing pattern from a lung patient extracted from a CyberKnife system (Accuray, Sunnyvale, CA) was programmed on the phantom. The amplitude and period (mean ± standard deviation) of the breathing pattern was 1.44±0.15 cm and 3.4±0.3 s, respectively.

An in‐house optical tracking system (OTS) was employed to validate the phantom motion. The OTS used a pair of CCD cameras (Polaris, Northern Digital Inc., Waterloo, Canada) to monitor four infrared reflective markers mounted on the moving insert of the Quasar phantom. The stability and tracking accuracy of the OTS was reported to be within 0.2 mm.^(31)^ The discrepancy between the motion pattern observed by the OTS and the reference pattern programmed on the Quasar phantom was evaluated for each respiratory pattern.

### B. Experimental data acquisition

#### B.1 Reference 4D CT data acquisition

A reference 4D CT dataset was used to evaluate the accuracy of the computed tumor trajectory in 4D CBCT (see Materials & Methods section D). A big bore 16‐slice CT scanner (Brilliance, Philips Healthcare, Andover, MA) was used to acquire the reference 4D CT scans of the Quasar phantom. The 4D CT scan was acquired with the standard parameters (120 kVp, 400 mAs/slice, 500 mm FOV, 0.5 s rotation time, 3 mm increment, and 3 mm slice thickness) for each respiratory pattern. A Bellows System (Philips Healthcare), strapped around the chest wall platform (linked to the moving insert) of the Quasar phantom, was used to provide the respiratory signal for phase sorting. The reconstructed 4D CT dataset consisted of 10 respiratory phases with equally spaced time intervals. The volumetric dataset corresponding to the end‐exhale phase was selected as the reference CT dataset. On the reference dataset, the target ball in the Quasar phantom was contoured as the gross tumor volume (GTV) which was subsequently used in the registration to compute the tumor trajectory in 4D CBCT (refer to Materials & Methods section D). The thresholding‐based automatic contouring tool in the Pinnacle treatment planning system (Philips Healthcare) was used to avoid the variations associated with manual target delineation. The threshold (CT number of −400) was chosen such that the computed target volume was closest to the known volume of the target ball. To evaluate the reproducibility of 4D CT reconstruction and GTV delineation, the 4D CT scan and automatic contouring procedure was repeated 10 times for each respiratory pattern.

#### B.2 4D CBCT data acquisition

Elekta Symmetry (Elekta, Norcross, GA) was used to acquire 3D and 4D CBCT scans of the Quasar phantom. Two 3D CBCT sequences (3D‐S for small FOV and 3D‐M for medium FOV) were used to acquire ground truth images. Details of the scanning sequences are listed in Table 1. In 4D CBCT, the system recorded projections with a dimension of 512×512 pixels at 5.5 frames per second. The projections were retrospectively sorted into 10 respiratory phases based on respiratory motion detected from moving anatomy (the target ball in our case) on 2D projections.^(32)^ The projections in each bin were then reconstructed using the Feldkamp–Davis–Kress (FDK) algorithm.^(33)^ A 4D CBCT scan of the Quasar phantom were firstly obtained with the manufacturer‐provided scanning sequence (4D‐S‐slow, see Table 1). The 4D‐S‐slow scan uses a small FOV with no bowtie filter (referred to as S20/F0) and deploys a slow scanning speed of $50^{\circ }/\min$ with a partial rotation ($∼200^{\circ }$). Four‐dimensional scans were also acquired with two modified imaging sequences (4D‐M‐slow and 4D‐M‐fast) by varying the FOV, the scanning speed, and the filter. Typically, the small FOV was employed for small anatomies, such as head and neck, while the medium FOV was required for large anatomies, such as chest and pelvis, to avoid the shading artifacts, image cutoff phenomenon, and truncated view. The 4D‐M‐slow and the 4D‐M‐fast scans used the medium FOV with a bowtie filter (referred to as M20/F1) by laterally shifting the detector panel. The slow scanning speed in the default setting increases the number of projections at the expense of longer scanning time and additional dose to the patient. To investigate the feasibility of reducing the scan time down to a practical level, we doubled the gantry rotation speed in our second modified sequence (4D‐M‐fast). Note that the imaging dose was proportional to the mAs used in the scan, which varied among sequences. The imaging dose measured with a farmer chamber (FC65‐P, Scanditronix Wellhofer, Bartlett, TN) placed at the center of a cylindrical acrylic phantom of 32 cm diameter is also listed in Table 1. A full 360° gantry rotation was used in both modified sequences, and all 4D CBCT images were reconstructed in a 270×264×270 voxels with a $1\;{\text{mm}}^{3}$ voxel size in this work.

**Table 1:** Scanning parameters for 3D CBCT and 4D CBCT data acquisitions

*Data Acquisitions*	*3D‐S*	*3D‐M*	*4D‐S‐slow*	*4D‐M‐slow*	*4D‐M‐fast*
Collimation/Filter	S20/F0	M20/F1	S20/F0	M20/F1	M20/F1
Beam area at iso (${cm}^{2}$)	27.7×27.7	42.6×27.7	27.7×27.7	42.6×27.7	42.6×27.7
Tube voltage (kVp)	120	120	120	120	120
Tube current (mA/ms per frame)	40/16	40/16	20/16	20/16	20/16
Number of projections (frames)	660	660	1320	2420	1270
Scan time (min)	2	2	4	7.2	3.6
Gantry rotation speed (°/min∼2 mm)	180	180	50	50	100
Gantry rotation (°)	−180∼180	−180∼180	−180∼20	−180∼180	−180∼180
Current time product (mAs)	429.8	423.7	420.8	773.1	405.8
Imaging dose (cGy)	1.01	0.62	0.99	1.14	0.60

### C. Image quality analysis

The image quality of 4D CBCT could be affected by several factors, of which two are the most important: 1) image artifacts (such as noise and streaking artifacts) mainly due to insufficient number of projections allocated in individual respiratory phases, and 2) the blurring caused by respiratory motion. In our case, the moving target inside the Quasar phantom supplied the respiratory signal for phase binning. It not only captured the motion blurring, but also carried the imaging artifacts induced by the respiratory signal and reconstruction algorithm, making it an ideal surrogate for image quality comparison among varying scanning sequences and respiratory patterns. In this study, image quality was assessed using signal‐to‐noise ratio (SNR), contrast‐to‐noise ratio (CNR), and motion blurring ratio (MBR) over the moving target in the Quasar phantom. Various periodic and aperiodic sinusoidal motions, as discussed in the Materials & Methods section A, were applied on the Quasar phantom for 4D CBCT scan. One‐way ANOVA test was used to examine if there was significant difference in the image quality between periodic and aperiodic breathing patterns.

#### C.1 Signal‐to‐noise ratio

**Figure 2:**
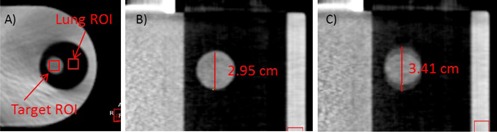
The selection of ROIs for SNR and CNR calculation (a). Shown here is the axial view of the slice bisecting the target ball in the Quasar phantom. The central coronal slices of the Quasar phantom (b) and (c), illustrating the MBR measurement for the end‐exhale phase and mid‐inhale phase, respectively.

SNR is a measure used to compare the level of a desired signal to the level of background noise inside a region of interest (ROI). In 4D CBCT, lower SNR indicates potentially higher noise and streaking artifacts. SNR was calculated as

(4) $$SNR=\frac{{\bar{x}}_{\text{target}}}{s_{\text{target}}}$$

where ${\bar{x}}_{target}$ and $s_{target}$ are the mean and the standard deviation of the pixel intensities inside a $7\times 7\;{\text{mm}}^{2}$ ROI in the moving target, respectively. Figure 2(a) illustrates the target ROI which was selected on the axial slice bisecting the target ball. The SNR was calculated for each of the 10 phases of the 4D CBCT images.

#### C.2 Contrast‐to‐noise ratio

CNR measures visibility of lung tumor in the presence of noise. Lower CNR indicates poorer tumor visibility. In this study, CNR was defined as

(5) $$CNR_{t\text{arg}et}=\frac{\left({\bar{x}}_{t\text{arg}et}-{\bar{x}}_{lung}\right)}{\sqrt{s_{t\text{arg}et}^{2}+s_{lung}^{2}}}$$

where ${\bar{x}}_{target}$ and ${\bar{x}}_{lung}$ are the mean pixel intensities of the target ROI and a $7\times 7\;{\text{mm}}^{2}$ lung ROI (see Fig. 2(a)), respectively; $s_{target}$ and ${\bar{s}}_{lung}$ are the standard deviations of the pixel intensities within the two ROIs, respectively. Note that the two ROIs were at the same horizontal level and ∼3.5 cm apart on the axial slice.

#### C.3 Motion blurring ratio

Four‐dimensional CBCT substantially reduces the motion blurring artifacts present in 3D CBCT. However, there is still residual motion blurring largely due to the phase‐based sorting algorithm in the commercial 4D CBCT system.^(12)^ We used MBR to quantify the residual motion blurring in 4D CBCT. On the coronal slice bisecting the target ball of the 4D CBCT scan, we measured the largest length of the target ball in the direction of the target motion. MBR was defined as the ratio between the measured length and the known diameter of the target ball (30 mm). Figures 2(b) and (c) illustrate the measurements. MBR was calculated for the end‐exhale phase and the mid‐inhale phase of 4D CBCT. The image for the end‐inhale phase showed similar image quality with the end‐exhale phase in the sinusoidal pattern and is not measured. In the end‐exhale phase, motion blurring directly affects our clinical decision regarding the sufficiency of target coverage. In the mid‐inhale phase, the worst motion blurring could be expected due to the nature of the phase‐based sorting algorithm.

### D. Analysis of target motion reconstruction in 4D CBCT

In lung SBRT, 4D CBCT is mainly used to calculate the time‐averaged target location and verify the sufficiency of treatment margin. Accuracy of target motion reconstruction in 4D CBCT is critical in these applications. In this study, we evaluated the accuracy of target motion reconstruction by tracking the center of gravity of the moving target on the reconstructed images in each phase. The trajectory of the moving target was then compared with the reference motion programmed on the Quasar phantom. Ideally, the target volume should be automatically delineated with the thresholding method described in Materials & Methods B.1. However, this method was not feasible due to the image noise and blurring artifacts in 4D CBCT.^(34)^ Manual contouring of the target volume was not ideal either, since uncertainties associated with manual contouring could compromise the robustness of our assessment.

**Figure 3:**
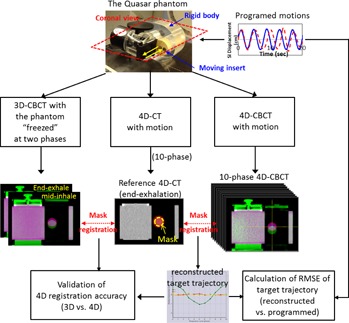
A schematic view of the workflow to validate the accuracy of the 4D CBCT registration by comparing with 3D CBCT registration, to compute the target trajectory in 4D CBCT and to calculate the RMSE of the reconstructed target trajectory.

Instead, we used a mask‐based automatic image registration method to compute the moving target trajectory. Figure 3 depicts our workflow. The basic idea was to first position the Quasar phantom with 3D CBCT, registering the rigid body of the phantom with the reference CT dataset (end‐exhalation phase of the 4D CT scan, see Materials & Methods B.1). This step eliminated residual setup errors. Then we acquired a 4D CBCT scan and registered each phase of the scan to the same reference CT dataset using the moving target only. A mask with 5 mm isotropic expansion around the GTV was created on the reference CT dataset for the registration. The registration was performed with grey value match of the mask region. The registration result (displacement of the target volume) on each phase of the 4D CBCT reconstructed the moving target trajectory. The grey value match within a mask region surrounding the GTV effectively aligned the centroid of the GTV with the center of the target ball on 4D CBCT, minimizing the influence of potential GTV shape change or noise and streaking artifacts caused by respiratory motion.^(35)^ By registering each phase image with the reference CT dataset, the moving target trajectory was reconstructed, as shown at the bottom of Fig. 3. Although the 4D CBCT scan for the breathing pattern exhibited significant motion blurring for mid‐inhale phases, the mask‐based 4D registration effectively aligned the centroid of the GTV in the reference CT dataset to the center of the target ball in the phase image of 4D CBCT, eliminating the influence of the motion blurring effect.

The accuracy of the mask‐based automatic image registration for 4D CBCT was validated by comparing with the 3D CBCT registration. The accuracy of the latter has been validated to be within sub‐mm.^(36)^ In this evaluation, we applied periodic motion patterns with four different periods (3, 4, 5, and 6 s) and three different amplitudes (10, 20, and 30 mm) on the Quasar phantom. Four‐dimensional CBCT scans were acquired with all three acquisition sequences, as listed in Table 1. Three‐dimensional CBCT scans were also acquired by "freezing" the moving target at either the end‐exhale or the mid‐inhale phase of the breathing cycle. The 3D CBCT scan and the 4D CBCT scan at these two phases were registered with the same reference CT dataset, using mask‐based automatic registration method. The difference between the registration results was evaluated to validate the method for target trajectory computation.

The extracted target trajectory was compared with the reference motion pattern applied on the Quasar phantom to quantify its accuracy. A root‐mean‐squared‐error (RMSE) was calculated by

(6) $$RMSE=\sqrt{\frac{1}{N}\sum_{n=1}^{N}{\left(s_{4DCBCT}(n)-S_{reference}(n)\right)}^{2},}$$

where *N* is the total number of respiratory phases, $s_{4DCBCT}(n)$ and $s_{reference}(n)$ are the extracted and reference target displacement for the nth phase, respectively. Since an aperiodic sinusoidal motion consisted of multiple breathing cycles with varying periods or amplitudes, a RMSE was calculated for each breathing cycle within the 4D CBCT scan. We reported the mean and the standard deviation of RMSEs over all the cycles. For periodic sinusoidal motion, the RMSEs over all cycles would be identical. In these cases, we reported the largest deviation (MaxE) between $s_{4DCBCT}(n)$ and $s_{reference}(n)$ over all phases in 1 cycle.

## III. RESULTS

As a measure of quality assurance on the breathing pattern applied on the Quasar phantom, we used an in‐house, high‐precision OTS to monitor the motion of the Quasar phantom during each CBCT scan. The maximum discrepancy in all cases was 0.2 mm, with an SD of 0.1 mm, suggesting excellent accuracy of the motion realized with the Quasar phantom.

### A. Reproducibility of 4D CT and automatic contouring

Table 2 shows the reproducibility of target localization at the end‐exhale phase in 4D CT for periodic sinusoidal motions with various periods and amplitudes. The standard Deviation of the centroid position of the GTV was calculated from the 10 repeated 4D CT scans. When the peak‐to‐peak amplitude was 10 mm, the SD was under 0.3 mm for all periods tested; when the amplitude was 20 or 30 mm, a trend of increase in standard deviation could be observed as the period increased. Overall, good reproducibility was found for the periodic sinusoidal motions with SDs being within 1 mm, validating the consistency of our method in localizing and contouring the GTV on 4D CT. This result is in line with the finding by Coolens et al.^(37)^ that the accuracy of 4D CT reconstructed motion amplitude was within submillimeter for sinusoidal breathing motions with 20–30 mm in amplitude and 3–6 s in period.

**Table 2:** Reproducibility of target localization and delineation at the end‐exhale phase of 4D CT

	*Standard Deviation (mm)*		
	*Peak‐to‐peak Amplitude*		
*Period (s)*	*10 mm*	*20 mm*	*30 mm*
3	0.25	0.58	0.40
4	0.26	0.53	0.75
5	0.17	0.99	0.89
6	0.29	1.03	0.91

### B. Analysis of image quality of 4D CBCT

**Figure 4:**
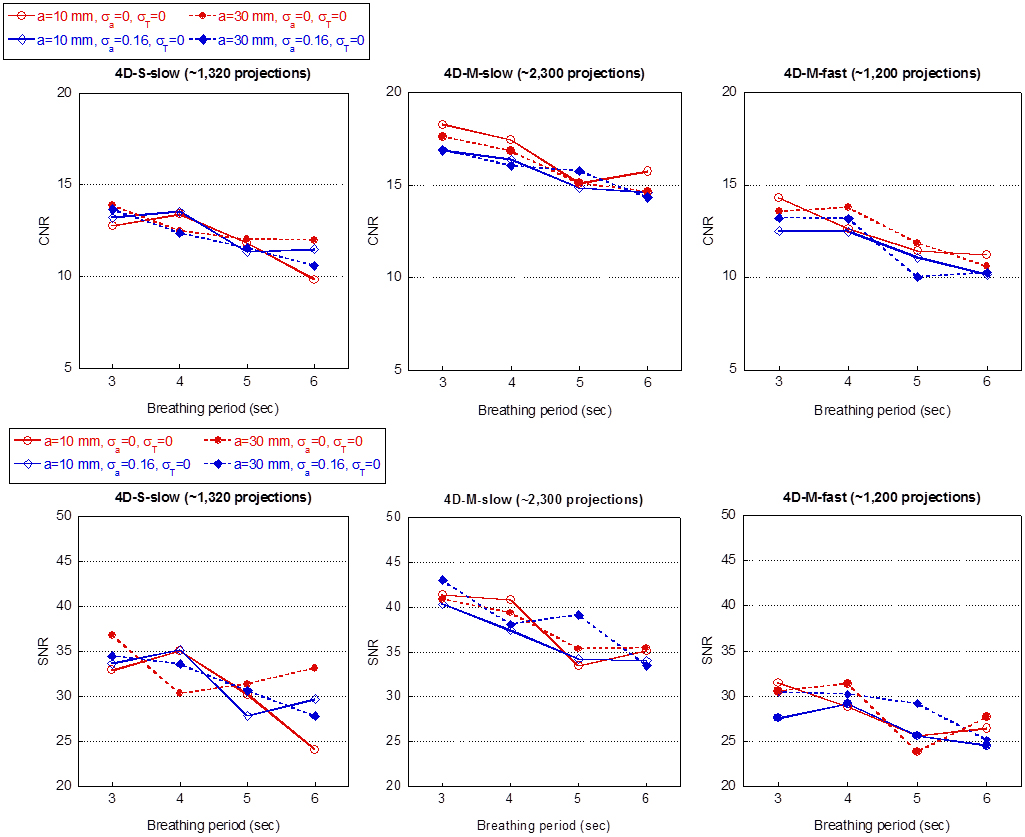
Mean CNR (top) and SNR (bottom) of 4D CBCT scans calculated over 10 respiratory phases with various breathing patterns applied. 4D CBCT scans were acquired with three different sequences: 4D‐S‐slow (left column), 4D‐M‐slow (middle column), and 4D‐M‐fast (right column).

Figure 4 shows the mean CNR and SNR values of the 4D CBCT images. The SDs of all data points ranged from 0.39 to 1.78 for CNR and from 2.04 to 6.59 for SNR and are not shown for reasons of clarity. The results for breathing patterns with amplitude of 20 mm are similar and not shown. The relative SD of the amplitude and period when applied was 0.16 and 0.70, respectively.

It was observed that the CNR values gradually decreased as the period increased from 3 to 6 s for all cases. The scans with the 4D‐M‐slow scan had higher CNR values than the scans with the other two 4D CBCT scan due to the larger number of projections used in reconstruction. The 4D‐S‐slow scan and the 4D‐M‐fast scan had similar number of projections and mAs. As a result, the CNR values were comparable between these two scans. No noticeable difference was observed between small and large amplitudes. The introduction of perturbation to the amplitude and the period of the breathing patterns (not shown in Fig. 4) had negligible effect on the CNR values. For example, in the one‐way ANOVA test comparing the CNR values for a periodic motion pattern and the two corresponding aperiodic motion patterns, when the amplitude was 10 mm, the p‐values were 0.86, 0.51, and 0.76 for 4D‐S‐slow, 4D‐M‐slow and 4D‐M‐fast, respectively; when the amplitude was 30 mm, the p‐values were 0.48, 0.93, and 0.77, respectively. The CNR values for the two 3D CBCT scans were comparable (25.87 for 3D‐S and 24.64 for 3D‐M). The larger FOV in the 3D‐M scan introduced more scatter in the scan; however, the effect of the scatter could have been partially compensated for by the use of the F1 filter.^(38)^

Similar trend was observed for the SNR values. The noticeable difference was the slightly larger decrease of the SNR values in the 4D‐M‐fast scan relative to the 4D‐S‐slow scan for all breathing patterns. The SNR values for the 3D‐S and 3D‐M scans were 64.93 and 60.72, respectively.

**Figure 5:**
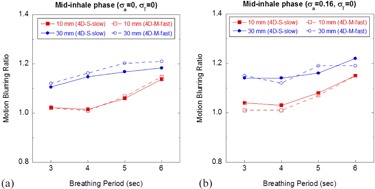
MBRs of 4D CBCT scans: (a) mid‐inhale phase with periodic motions, and (b) mid‐inhale phase with aperiodic motions.

Figure 5 shows the MBRs for the mid‐inhale phases of 4D CBCT. All measurements were done with the same window level and width setting. The difference among the MBRs for the three scanning sequences was negligible and the results for 4D‐M‐slow are not shown. The motion blurring at the end‐exhale phase for all studied breathing patterns was negligible, with the MBRs being close to unit. In general, more pronounced motion blurring was observed at the mid‐inhale phase than at the end‐exhale phase. For the mid‐inhale phase as shown in Fig. 5, there was a strong dependence of the MBRs on the base amplitude as larger MBRs were observed for the motions with larger amplitude. It was also observed that the MBRs increased as the period increased. The worst motion blurring (MBR=∼1.2) was observed at the mid‐inhale phase for the motion pattern with 30 mm amplitude and 6 s period (Fig. 5(b)), indicating the length of the target ball in the motion direction was increased by ∼6 mm.

For the real patient breathing pattern, the CNR/SNR with 4D‐S‐slow, 4D‐M‐slow, and 4D‐M‐fast was 14.34/36.22, 17.00/41.37, and 15.43/31.46, respectively. While the MBRs for end‐exhale phase with all three scanning sequences were close to unity, the MBRs for mid‐inhale phase were 1.07, 1.07, and 1.08 with 4D‐S‐slow, 4D‐M‐slow, and 4D‐M‐fast, respectively. These results fell into respective ranges of the data with simulated breathing patterns.

### C. Evaluation of extracted target motion accuracy

Table 3 shows the accuracy of the 4D CBCT image registration as compared with the 3D CBCT registration. In general, less discrepancy was observed at mid‐inhale phase than at end‐exhale phase, despite worse motion blurring (larger MBR) associated with the former, especially when the amplitude was large. When the amplitude was 20 mm or less, the discrepancy was within 0.5 mm for both phases; for 30 mm amplitude, the error at the mid‐phase was within 0.1 mm, while the error at the end‐exhale phase ranged from 0.7 mm to 1.0 mm. Our results agree with that of Sonke et al.^(30)^ who used a similar method to validate the accuracy of 4D CBCT registration. No dependency on the period of the breathing pattern or the scanning sequence was observed.

**Table 3:** Accuracy of the 4D CBCT registration method evaluated at mid‐inhale and end‐exhale phases. Shown in the table are the deviations between 4D CBCT registration and 3D CBCT registration

		*Scanning Sequences*											
		*4D‐S‐slow (mm)*				*4D‐M‐slow (mm)*				*4D‐M‐fast (mm)*			
*Amp. (mm)*	*Position*	*3s*	*4s*	*5s*	*6s*	*3s*	*4s*	*5s*	*6s*	*3s*	*4s*	*5s*	*6s*
10	mid‐inhale	0.1∼0.2				0.1∼0.2				0.0∼0.1			
	end‐exhale	0.1∼0.2				0.1∼0.3				0.1 ∼0.4			
20	mid‐inhale	0.1∼0.3				0.0∼0.1				0.0			
	end‐exhale	0.2∼0.4				0.4∼0.5				0.4∼0.5			
30	mid‐inhale	0.1				0.0∼0.1				0.0∼0.1			
	end‐exhale	0.7∼0.8				0.9∼1.0				0.9∼1.0			

**Figure 6:**
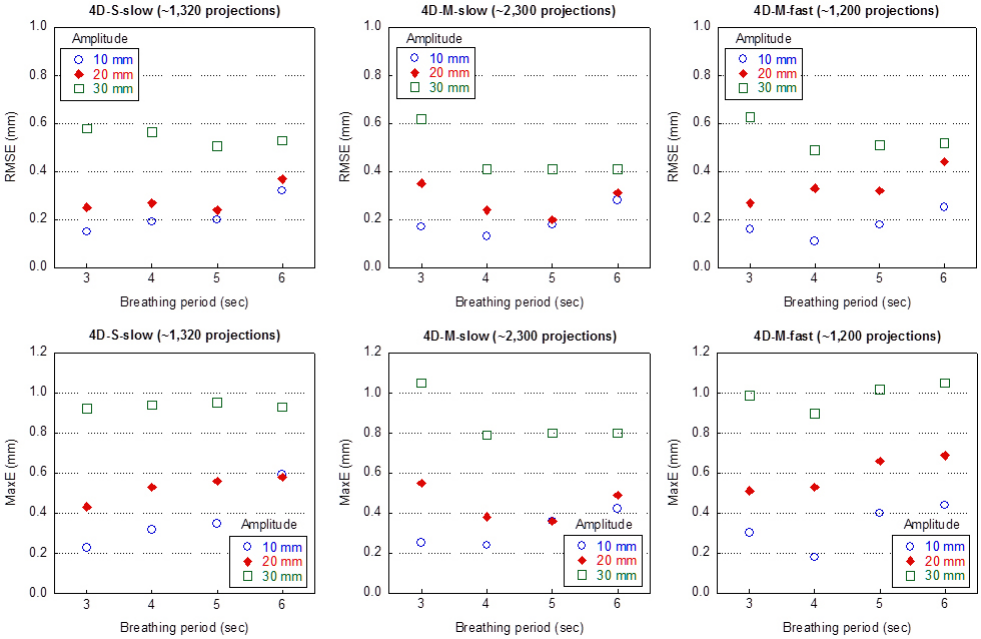
Accuracy of tumor motion reconstruction of 4D CBCT for periodic breathing patterns. RMSE and MaxE were calculated over the 10 phases of the 4D CBCT scan in one breathing cycle. For each breathing pattern, 4D CBCT scans were obtained with three different scanning sequences.

Figure 6 shows the RMSE and the MaxE values of the extracted target motion trajectory from 4D CBCT with periodic signal of various periods and amplitudes applied on the Quasar phantom. In general, the RMSE increased slightly (<0.5 mm) when the amplitude increased from 10 mm to 30 mm. For all periodic breathing patterns tested, the RMSEs were within 0.7 mm. No dependence of the RMSE on the breathing period could be observed. The RMSEs were comparable among all three scanning sequences. The MaxE showed similar trend, with the largest MaxE being around 1.0 mm when the amplitude was 30 mm.

**Figure 7:**
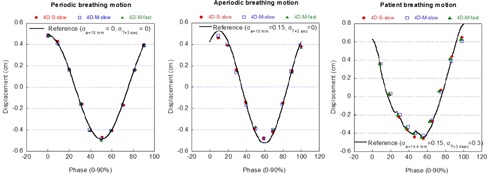
Comparison between extracted motion trajectories from 4D CBCT and references: periodic breathing pattern (left column), aperiodic breathing pattern (middle column), and real patient breathing pattern (right column).

Figure 7 shows representative examples of the target motion trajectories reconstructed from 4D CBCT, superimposed over respective reference breathing patterns: a periodic breathing motion with 10 mm amplitude and 3 s period, an aperiodic breathing motion with 10 mm amplitude and 3 sec period with cycle‐to‐cycle variation in amplitude ($\sigma_{a}=0.16$), and the actual patient breathing pattern. A single cycle was randomly selected from the aperiodic breathing pattern and the actual patient breathing pattern for demonstration. These examples demonstrated that the reconstructed target motion trajectories had comparable accuracy among all three scanning sequences.

**Figure 8:**
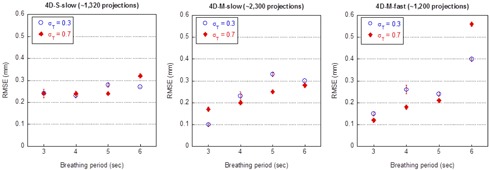
Accuracy of tumor motion reconstruction of 4D CBCT for aperiodic breathing patterns with 10 mm amplitude and cycle‐to‐cycle variation in period. A RMSE value was calculated over the 10 phases of each cycle. Shown in the figure is the mean and standard deviation (SD) of RMSEs over all cycles.

Figure 8 shows the mean and the standard deviation of the RMSE values of the extracted motion trajectories from the 4D CBCT images acquired for aperiodic motion patterns with varying periods (relative $\text{SD}\;\sigma_{T}=0.3\;\text{or}\;0.7$). The amplitude of the signal remained constant during each scan. Shown in the figure are the results for aperiodic motion patterns with 10 mm amplitude. The results for motion patterns with 20 and 30 mm amplitude were very similar and are not shown. The mean RMSE values in all cases were generally within 0.3 mm, with the exception for the signal with 6 s period scanned with the 4D‐M‐fast scan, where the maximum was 0.40 and 0.56 mm for $\sigma_{T}=0.3$ and 0.7, respectively. The differences between $\sigma_{T}=0.3$ and 0.7 were within 0.2 mm for all breathing periods with all three scans and no strong dependence of mean RMSE on $\sigma_{T}$ was observed. The small standard deviations (<0.02 mm for all breathing patterns) of the RMSE values indicated that the impact of the variation of the period on the accuracy of tumor motion trajectory reconstruction was negligible.

**Figure 9:**
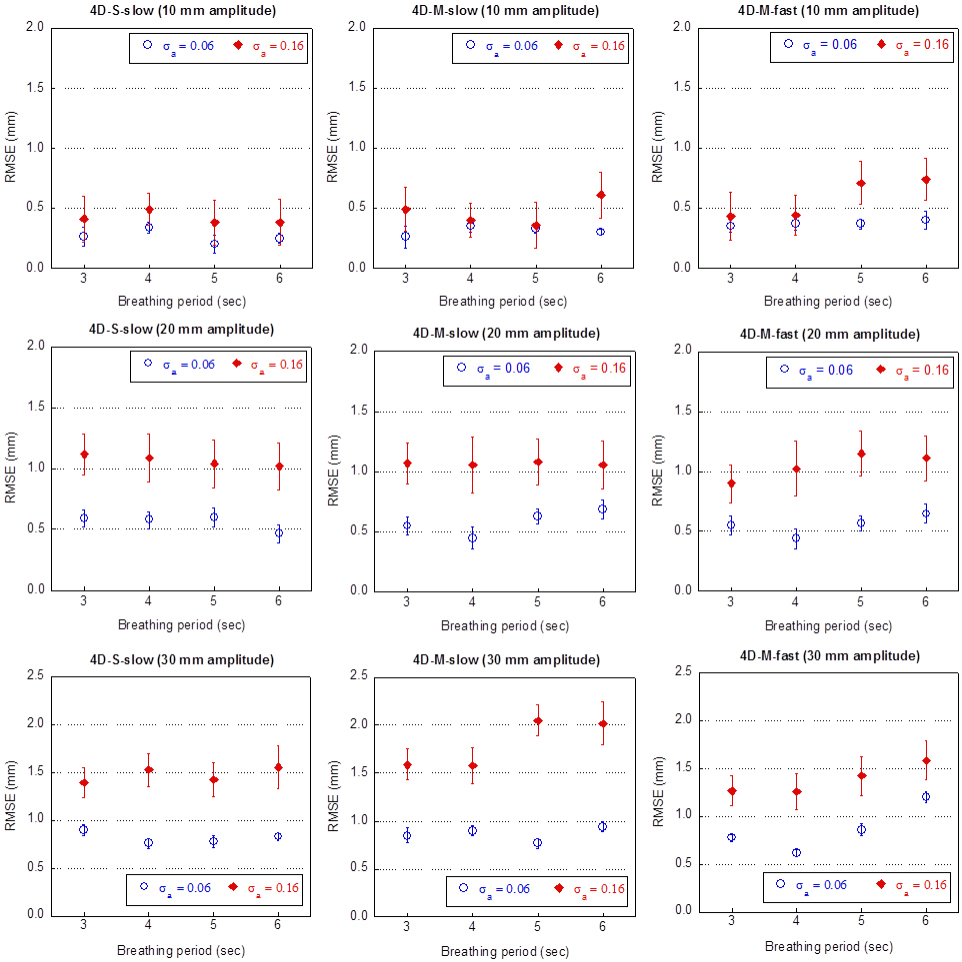
Accuracy of tumor motion reconstruction of 4D CBCT for aperiodic breathing patterns with cycle‐to‐cycle variation in amplitude. A RMSE value was calculated over the 10 phases of each cycle. Shown in the figure is the mean and standard deviation (SD) of RMSEs over all cycles.

Figure 9 shows the mean and standard deviation of the RMSE values of the extracted motion trajectories from the 4D CBCT images acquired for aperiodic motion patterns with varying amplitudes ($\sigma_{a}=0.06\;\text{or}\;0.16$). The period of the signal remained constant during each scan. While the mean RMSE showed no dependence on the period of the breathing pattern, a strong dependence on the amplitude could be observed. When the amplitude of the signal increased from 10 mm to 30 mm, the mean RMSE increased on average, for all three scanning sequences, by 0.5 and 1.0 mm for $\sigma_{a}=0.06\;\text{and}\;0.16$, respectively. The difference between the mean RMSE values for $\sigma_{a}=0.06\;\text{and}\;0.16$ increased as the amplitude increased as well: with $\sigma_{a}=0.16$, the mean RMSE values were on average greater than those with $\sigma_{a}=0.06$ by 0.2, 0.5 and 0.8 mm when the amplitude was 10, 20, and 30 mm, respectively. The maximum of the mean RMSE values over all the cases was slightly over 2.0 mm when period = 5 or 6 s and amplitude = 30 mm and the 4D‐M‐slow scan was used. The standard deviation of the RMSE values remained consistent in all cases and was close to the amount of perturbation introduced to the amplitude (0.06 when $\sigma_{a}=0.06$ and 0.18 when $\sigma_{a}=0.16$). Since the mean RMSE values were comparable among the three scanning sequences for each respective breathing pattern, we concluded that there was no dependence of the mean RMSE values on the scanning sequences.

For the real patient breathing pattern, the RMSE (mean ± standard deviation) for 4D‐S‐slow, 4D‐M‐slow, and 4D‐M‐fast was 0.71±0.18,0.78±0.18, and 0.81±0.15 mm, respectively; the MaxE for 4D‐S‐slow, 4D‐M‐slow, and 4D‐M‐fast was 1.28±0.32, 1.38±0.31, and 1.51±0.26 mm, respectively.

## IV. DISCUSSION

In this work, we conducted a comprehensive study of the performance of the 4D CBCT system. It is worth emphasizing that, unlike a lot of studies on 4D CBCT within research settings, we strive to quantify a commercially available 4D CBCT system and to expand its clinical use. The vendor‐supplied acquisition sequence (4D‐S‐slow) utilizes a partial gantry rotation with slow gantry motion ($200^{\circ },50^{\circ }/\min$) and small collimation (FOV: 27.7 cm in transverse direction and 27.7 cm in the SI direction). Four‐dimensional CBCT scans with this sequence suffer from volume truncation effect due to limited field of view for clinical imaging, which motivates us to adapt the scanning parameters for better image quality. Based on our clinical experience with 3D CBCT, M20 collimation with F1 filter is needed for typical clinical volumes involving thorax which increases the FOV in the transverse direction to 42.6 cm. Our modified scanning sequences also utilize a full gantry rotation (360°) to provide more projections in the hope to enhance image quality. But due to the slow gantry rotation speed ($50^{\circ }/\min$), the scanning time and imaging dose increase significantly as well (see Table 1, 4D‐M‐slow). To address this issue, in our second modified scanning sequence (4D‐M‐fast), we double the gantry rotation speed ($100^{\circ }/\min$) to reduce the scanning time to an acceptable range and the imaging dose to the same level as a 3D CBCT scan. It is worth pointing out that there are potentially sets of scanning parameters which can reach better trade‐off between image quality, tumor localization accuracy, imaging dose, and acquisition time than the sequences studied here. The search for the optimal scanning sequence is beyond the scope of this work.

The impact of the scanning sequences on image quality and, most importantly, the ability of the system in accurately reconstructing tumor motion trajectory, need to be assessed, especially, for irregular breathing motion patterns. We used a respiratory motion phantom for this purpose. The advantage of using a phantom (instead of a patient) is that we can perform 4D CBCT scan repeatedly with different combinations of scanning sequence and breathing motion. The performance of the 4D CBCT system under various conditions can be compared with a common criterion standard (i.e., the image quality of the 3D CBCT scan in image quality assessment and the programmed tumor motion in tumor motion reconstruction accuracy evaluation). We found the Quasar phantom with a target ball embedded inside material with density equivalent to lung suitable for our purpose, mimicking a mobile lung tumor for which 4D CBCT is most useful.

As a first step to evaluate the performance of 4D CBCT, we used SNR and CNR to quantify its image quality, which have been widely used for CBCT image quality quantification.^18^, ^39^ While SNR quantifies the signal level in the presence of noise, CNR indicates the capability of the system in discerning fine details of small objects. Generally, SNR and CNR are proportional to the number of projections used in the reconstruction and linear with the tube current. Thus, compared with 3D CBCT, the large drop of both SNR and CNR in 4D CBCT can be attributed to the facts that the tube current is reduced by half and each phase of the 4D CBCT only uses one‐tenth of the total projections for reconstruction. Similarly, 4D‐M‐slow scan showed better CNR and SNR than 4D‐S‐slow, in spite of increased scatters from increased collimation. The noise induced by increased scatters was compensated for by the reconstruction using double number of projections. Additionally, unlike 4D‐S‐slow scan which only uses a 200° partial arc, 4D‐M‐slow scan uses a full 360° gantry rotation which spreads out the projections. The FDK algorithm is known to perform better when the projections are evenly spread out surrounding the objects.^(20)^ The image quality degradation from 4D‐M‐slow scan to 4D‐M‐fast scan can be explained by the reduced number of projections and increased likelihood of missing projections due to faster gantry rotation in 4D‐M‐fast scan.^(40)^ However, it is interesting to observe that the image quality of the 4D‐M‐fast scan was comparable with that of the 4D‐S‐slow scan. For all three 4D CBCT scanning sequences, the impact of cycle‐to‐cycle variation in either amplitude or period of the breathing pattern on both CNR and SNR was small and no clear trend was observed. Likewise, no dependence of either CNR or SNR on the amplitude of the motion was noticed. However, as breathing period increased, both SNR and CNR decreased in all three scanning sequences. As breathing period increased, the 4D CBCT scan included fewer breathing cycles, which resulted in increased angular gap between the clusters of projections belong to the same phase, thereby degrading image quality. Image quality variation among respiratory phases (interphase variations) was noticed; however, no specific trend was observed for the simulated motions. This might be different in real patient's breathing because patients can occasionally present large baseline shifts or long‐lasting end exhalation phase. As a result, the lack of projections or uneven angular gap between projections can cause more pronounced image quality degradation, thus, interphase variations.^(18)^

We also used MBR to evaluate the residual motion blurring in 4D CBCT, which basically quantified the shape change (elongation) of the target along the motion direction. As shown in Fig. 5, significantly larger motion blurring was observed at mid‐inhale phase than at end‐exhale phase, which can be explained by examining the 4D binning algorithm used in the commercial system. Though the respiratory signal is recovered using the Amsterdam shroud method based on the motion amplitude, a phase‐based sorting algorithm is used to place individual projections into respective phase bins.^12^, ^32^ Thus, at mid‐inhale phase, the target travels a larger distance than at end‐exhale phase, causing larger motion blurring on the phase images. The larger the amplitude, the worse the motion blurring, as is seen in Fig. 5. At the mid‐inhale phase, the image quality degradation due to the increase in breathing period played a significant role in the motion blurring, especially for breathing motion with small amplitude (10 mm), causing the MBR to gradually increase. The cycle‐to‐cycle variation on the amplitude worsened the motion blurring regardless of the amplitude of the breathing motion (Fig. 5(b)). It is interesting to notice the remarkable agreement of the MBRs between the 4D‐S‐slow and 4D‐M‐fast scans.

While the standard image quality metrics can be used to assess the image quality of 4D CBCT which correlates well with our ability in precisely localizing the tumor, in this work, we directly evaluated tumor localization accuracy on individual phases, thus the accuracy of tumor motion trajectory reconstruction, which is of utmost interest in the clinical use of 4D CBCT. To that end, we used the automatic registration function in Elekta Symmetry which registered individual 4D CBCT phases with a reference CT image set. Accuracy of the 4D CBCT registration was validated with 3D CBCT registration at two phases (mid‐inhale phase and end‐exhale phase) by manually "freezing" the target ball at corresponding positions. Slightly larger discrepancy was observed at the end‐exhale phase with large amplitude due to the uncertainty in manually "freezing" the target ball close to the extreme of its moving range, but overall accuracy was within submillimeter. The design of the Quasar phantom allows one to manually position the target ball within a ± 30 mm range; however, it is more accurate to position it at the center than at the extreme position. Note the uncertainty was not a concern when the respiratory phantom performed sinusoidal motion, as evidenced by the excellent accuracy of target ball positioning monitored with a high‐precision OTS. Our results show that when there was no perturbation in cycle‐to‐cycle amplitude perturbation, the accuracy of tumor motion trajectory reconstruction was within 1 mm regardless of the scanning sequence. However, the perturbation of cycle‐to‐cycle amplitude caused RMSE to increase, especially when the nominal amplitude was large. When the nominal amplitude was 30 mm and $\sigma_{a}=0.16$, RMSE was ∼2 mm regardless of the scanning sequence. It is interesting to note that the RMSE increased with the introduction of amplitude perturbation, while the standard image quality metrics such as SNR and CNR were not affected. It is also interesting to note that, when the breathing period increases, there is noticeable image quality degradation but no impact on the tumor motion trajectory accuracy, which may be due to the mask‐based 4D CBCT image registration which aligned the center of gravity of the target ball on two images. These findings suggest that, while quantitative assessment of image quality is important, it is equally important to directly examine the system's ability in localizing tumor on individual phases.

Our results show that with the 4D‐M‐fast scanning sequence, comparable image quality and tumor motion reconstruction accuracy can be achieved as the 4D‐S‐slow scan regardless of the respiratory motion pattern, suggesting that it is suitable for clinical use. Three‐dimensional CBCT scan suffers from severe respiration‐induced motion blurring, while the 4D‐S‐slow scan suffers from volume truncation artifacts due to its limited FOV. Our study demonstrated that the vendor‐supplied scanning parameters for 4D CBCT can be modified by using a medium FOV to acquire images with quality similar (albeit lower) to that of 3D CBCT (3D‐M) but without severe respiratory blurring, while the image dose can be maintained similar to that of conventional 3D CBCT. Significant reduced motion blurring in 4D CBCT gives it advantage over 3D CBCT in clinical applications, such as determining the sufficiency of treatment margin or calculating time‐averaged mean tumor position for localization.

One limitation of our study is that we used sinusoidal waves to simulate patient breathing pattern and used only one real patient's breathing pattern. The perturbation on cycle‐to‐cycle amplitude and period is a simplification of the variation within a real patient's breathing pattern. Although the results for the real patient's breathing pattern were found to fall into respective ranges obtained with simulated breathing patterns, larger interphase variations can be expected when patients present irregular breathing pattern. In our future study, we plan to collect more patient breathing patterns and apply on the Quasar phantom for further investigation. Another limitation is the calculation of the MBR which uses a subjective measurement. The current system does not support image export for 4D CBCT which limits our ability in performing an objective calculation. However, the window and level was adjusted to the same setting for all measurements. And the remarkable agreement of the MBRs between the 4D‐S‐slow and 4D‐F‐fast suggests the validity of our method for cross‐comparison.

## V. CONCLUSIONS

Four‐dimensional CBCT alleviated motion blurring artifacts at the cost of degraded image quality, as compared with 3D CBCT. The manufacture‐supplied scanning sequence (4D‐S‐slow) suffered additional volume truncation artifacts due to the use of the small FOV when imaging lung patient for SBRT. Our results indicated that the medium FOV can be utilized to reduce such artifacts by increasing the scanning speed. Slight loss in image quality with the new sequence (4D‐M‐fast) was observed; however, the accuracy in localizing the mobile target was not affected. The irregularity in the breathing pattern simulated in this study increased blurring, but had negligible effect on the image quality of 4D CBCT in terms of CNR and SNR. It slightly reduced the accuracy of motion trajectory reconstruction. Nevertheless, the overall accuracy in tumor motion reconstruction was ∼1 mm when the motion amplitude was less than 20 mm and ∼2 mm when the amplitude was 30 mm. Our findings suggest that the 4D‐M‐fast scanning sequence is suitable for clinical use in lung SBRT.
